# Actinomycosis in a Dog

**Published:** 1902-04

**Authors:** H. W. Gohn

**Affiliations:** St. John’s, Mich.


### ACTINOMYCOSIS IN A DOG.

By H. W. Gohn, V.S.,
ST. JOHN'S, MICH.

I was called to see a mastiff that had been bitten over the upper third of the tibia. The injury did not seem severe, and mild antiseptic treatment was given, with the result that recovery seemed rapid, and no more was heard of the case until six weeks later, when the animal was brought to me with considerable swelling of the parts and pus discharging from four or five openings. In order to inject these tracts it was necessary to enlarge some of the openings where the new-growth was found to be of a very firm nature. Deciding to use a caustic, I injected once daily, for four or five days, a dilution of hydrochloric acid, after which it healed promptly, with little thickening. However, a few weeks later the owner reported the animal as having some warts in his mouth, and when I again examined him I found the mucous membrane of the mouth covered with almost innumerable growths, which varied in size from that of a pin-head beneath the membrane to those which projected from it the size of a bean, while from the corners of the mouth they hung in a mass resembling a cluster of grapes.

The tongue was covered, and on opening the mouth wide there was no visible mucous membrane that was not studded with these growths. This condition was accompanied with a very fetid odor. Securing the animal, I removed one of the growths and had it examined microscopically, which proved it to be actinomycotic.

The treatment given was potassium iodide internally, and a good recovery resulted, with no recurrences in several years, during which I frequently saw the animal.

I was slow to pronounce the case actinomycosis before the microscopical examination, as I had never seen it mentioned in any veterinary work. It was, however, suggested by Steele, in his treatise on *The Ox* (p. 140). Since the treatment of this case, however, I

have found mention of one in Friedberger and Frohner's *Pathology* and one case reported in the columns of the JOURNAL. The inoculation must have occurred at the time of the first injury or soon after, developing in due time the suppurating tracts from which, by licking, the animal inoculated the fungus into the mucous membrane of the mouth.

